# Serum pentraxin-3 as a potential biomarker for diagnosis and prognosis in primary liver cancer: An observational study

**DOI:** 10.1097/MD.0000000000040421

**Published:** 2024-12-13

**Authors:** Li Chen, Shifu Song, Mao Chen, Qin Liu, Hongchi Zhou

**Affiliations:** a Department of Radiology, Clinical Medical College and The First Affiliated Hospital of Chengdu Medical College, Chengdu, China; b Department of Emergency Medicine, Clinical Medical College and The First Affiliated Hospital of Chengdu Medical College, Chengdu, China; c Department of Hepatobiliary and Vascular Surgery, Clinical Medical College and The First Affiliated Hospital of Chengdu Medical College, Chengdu, China.

**Keywords:** AFP, biomarker, CA199, pentraxin 3, primary liver cancer

## Abstract

This study aimed to examine serum pentraxin 3 levels in patients with primary liver cancer and to assess its potential as a diagnostic and prognostic biomarker. Serum samples were obtained from 180 patients with primary liver cancer and 180 healthy control subjects. The concentration of PTX3 in these samples was measured using an ELISA kit. The study also investigated the correlation between PTX3 levels and the clinicopathological characteristics of patients with primary liver cancer. The effectiveness of serum PTX3 in diagnosing primary liver cancer was evaluated using receiver operating characteristic (ROC) curves and their corresponding areas under the curve (AUC). The prognostic significance of serum PTX3 in patients with primary liver cancer was assessed using Kaplan–Meier survival curves. Serum PTX3 levels were elevated in patients with primary liver cancer compared to those in healthy control subjects. These levels were significantly correlated with drinking history, TNM stage, BCLC stage, tumor size, tumor number, and vascular invasion. However, no significant correlations were observed between PTX3 levels and other factors, such as age, sex, BMI, liver cirrhosis, histological grade, and histological type. With a cut-off value of 5.1 ng/mL, PTX3 effectively differentiated patients with primary liver cancer from healthy control subjects, achieving an AUC of 0.734, a sensitivity of 73.24%, and a specificity of 84.78%. Patients with higher serum PTX3 levels had lower overall survival rates and recurrence-free survival rates than those with lower PTX3 levels. Serum PTX3 levels are elevated in patients with primary liver cancer and high serum PTX3 levels are associated with poor prognosis. This suggests that serum PTX3 has the potential to be a novel biomarker for both the diagnosis and prognosis of primary liver cancer. These findings may improve patient outcomes by enabling early detection and continuous monitoring.

## 1. Introduction

Liver cancer is the sixth most prevalent malignant tumor and the third leading cause of cancer-related death worldwide.^[1]^ Despite surgical resection being the primary treatment for liver cancer, the postsurgical recurrence rate exceeds 70% and the 5-year survival rate remains <8%.^[2,3]^ Typically diagnosed at intermediate or advanced stages, most liver cancer cases are not amenable to curative surgical treatment, leading to both high recurrence and low survival rates.^[4]^ This underscores the urgent need for early screening strategies to improve diagnosis and ensure timely treatment.

Currently, AFP is the most widely used biomarker for liver cancer screening and diagnosis, with sensitivity and specificity for hepatocellular carcinoma ranging from 41% to 65% and 80% to 94%, respectively.^[5,6]^ However, about 40% of hepatocellular carcinoma patients do not have elevated AFP levels, and chronic liver diseases can also result in elevated AFP levels during active inflammation.^[7,8]^ Additionally, AFP has limited sensitivity to intrahepatic cholangiocarcinoma.^[9,10]^ These findings suggest that AFP has limitations in diagnosing liver cancer.

Pentraxin-3 (PTX3), a glycoprotein secreted by immune cells, plays an important role in inflammation, immune responses, apoptosis, and vascular remodeling.^[11,12]^ PTX3 promotes cellular proliferation, angiogenesis, and resistance to apoptosis, facilitating cancer cell invasion and growth via the AKT and NF-κB signaling pathways.^[11]^ It also helps cancer cells evade immunosurveillance. Emerging evidence indicates that serum PTX3 levels are elevated in several types of cancers and may correlate with disease progression and prognosis.^[13,14]^ However, its role in primary liver cancer remains unclear. This study aimed to investigate the potential of serum PTX3 as a biomarker for the diagnosis and prognosis of primary liver cancer. By examining the association between serum PTX3 levels and clinical outcomes in patients with primary liver cancer, we hope to explore its potential utility in clinical practice.

This study presents a comprehensive analysis of serum PTX3 levels in patients with primary liver cancer and evaluates its diagnostic and prognostic significance. Our findings may contribute to the development of more effective strategies for early detection and management of primary liver cancer, ultimately improving patient survival and quality of life.

## 2. Patients and methods

### 2.1. Study population

This study was approved by the Ethics Committee of The First Affiliated Hospital of Chengdu Medical College (No. 21423715). All the participants provided written informed consent. Patients who were newly diagnosed with primary liver cancer between January 2019 and December 2020 were recruited. The inclusion criteria were as follows: patients diagnosed with primary liver cancer by histopathological examination of surgical resection specimens or imaging criteria; no prior treatment, including surgery, radiotherapy, or chemotherapy; absence of other malignant tumors; and complete patient record information. The exclusion criteria included patients with the following conditions: malignancies other than primary liver cancer and acute or chronic inflammation.

We used the Power Analysis and Sample Size (PASS) software to calculate the statistical power, effect size, and minimum sample size required for the study. The minimum sample size was determined to be 168. However, we included a total of 360 study participants, comprising 180 primary liver cancer patients and 180 healthy control subjects. Clinical and demographic data of the study population were obtained from the electronic medical record system. The drinking history was based on a previously described questionnaire that assessed self-reported alcohol consumption patterns.^[15,16]^

### 2.2. Diagnosis of primary liver cancer

The diagnosis of primary liver cancer was based on clinical manifestations, MRI and/or CT scans, serum AFP and CA199, and histopathological examinations of surgical resection specimens.

### 2.3. Measurement of serum PTX3 levels

Serum PTX3 levels in liver cancer patients and control subjects were assessed using a commercially available ELISA kit (Abcam, Shanghai, China) following the manufacturer’s instructions. Briefly, 96-well ELISA microplates containing PTX3 antibody were washed 3 times with PBST, and then 300 μL of blocking buffer was added. The plates were maintained at room temperature for 1 hour. Subsequently, the blocking buffer was removed, 100 μL of serum samples were added to each well, with 100 μL of PBS used as a negative control. The microplates were then incubated at room temperature for 2 hours. After washing the plates with PBST 3 times, 100 μL of the detection antibody (0.5 μg/mL) was added and kept at room temperature for 2 hours. The microplates were added with 100 μL of avidin-HRP-conjugated secondary antibody in each well and kept at room temperature for 20 minutes. Following 3 washes with PBST, 100 μL of TMB substrate solution was added to each well of the microplate. The plates were kept at room temperature for 20 minutes to allow chromogenic reactions. The wells were added with 50 μL of Stop Solution to stop the chromogenic reaction. A Model 680 microplate reader was used to measure absorbance at 450 nm. Each serum sample was analyzed in triplicate.

### 2.4. Serum assays for AFP and CA199

Serum levels of AFP and CA199 were quantified using the Roche Cobas E601 immunoassay analyzer through electrochemiluminescence immunoassays, employing specialized reagents, and strictly following the manufacturer’s guidelines. These assays were performed at the Department of Laboratory Medicine, The First Affiliated Hospital of Chengdu Medical College.

### 2.5. Immunohistochemistry

Tissue samples were fixed in 4% paraformaldehyde solution for 48 hours. Subsequently, the tissue samples were embedded in paraffin and sliced into 4 µm thick sections. The sections were subjected to dewaxing, dehydration, and antigen retrieval. After washing 3 times with PBST, the sections were treated with 10% goat serum to block nonspecific staining. The sections were stained with an anti-PTX3 antibody from Abcam, while the negative controls were not exposed to the primary antibody. After 3 washes with PBS, the sections were stained with a secondary antibody. Following 3 additional washes with PBS, color development was achieved using diaminobenzidine. Pathologists analyzed PTX3 expression using a Zeiss LSM500 microscope.

### 2.6. Patient follow-up

Patient follow-up included clinical evaluations for liver decompensation and the early detection of recurrence via MRI or CT scans, following NCCN and ESMO guidelines.^[17,18]^ Multiphasic, high-quality, cross-sectional MRI or CT scans of the chest, abdomen, and pelvis were performed every 3 to 6 months for the first 2 years, and then every 6 months thereafter. Serum AFP levels were measured every 3 to 6 months for the first 2 years, and then every 6 months for at least 5-years.

Overall survival (OS) was defined as the time from the initial diagnosis of liver cancer to either death from any cause or the last follow-up date. Recurrence-free survival (RFS) was defined as the period during which liver cancer patients remained disease-free or survived without recurrence after treatment.

### 2.7. Statistical analysis

SPSS software was used for statistical analyses. The independent-sample *t*-test or Wilcoxon rank-sum test was used to evaluate the statistical significance of continuous variables, while the chi-square test or Fisher exact test was used for categorical variables. The diagnostic performance of serum PTX3 as a biomarker for liver cancer was assessed using the receiver operating characteristic (ROC) curve and the corresponding area under the curve (AUC). Survival differences were analyzed using Kaplan–Meier survival curves and log-rank tests. A *P*-value of <.05 was considered statistically significant.

## 3. Results

### 3.1. Clinical profiles and serum PTX3 concentrations in the study population

This study included a cohort of 180 patients diagnosed with primary liver cancer, consisting of 145 individuals with hepatocellular carcinoma and 35 with intrahepatic cholangiocarcinoma, as well as 180 healthy control subjects. There were no statistical significant differences between the 2 groups regarding age, height, gender, weight, HCV status, or BMI. However, significant differences were observed between the 2 groups in terms of drinking history and HBV status. Furthermore, the levels of PTX3, AFP, and CA199 were significantly elevated in the liver cancer group compared to the healthy control group (*P < *.05) (Table 1).

**Table 1:** Clinical profiles and serum PTX3 concentrations in the study population.

Characteristics	Liver cancer patients	Healthy control subjects	*P*-value
Age (yr)	64.2 ± 6.2	65.6 ± 5.2	.231
Gender			
Male	127	115	.178
Female	53	65	
Height (cm)	171 ± 7.3	167 ± 5.9	.316
Weight (kg)	63.3 ± 5.2	66.7 ± 5.2	.217
BMI (kg/m^2^)	25.4 ± 3.7	26.8 ± 4.1	.132
Drinking history			
Yes	132	52	<**.0001**
No	48	128	
HBV status			
Positive	125	44	<**.0001**
Negative	55	136	
HCV status			
Positive	12	9	.49
Negative	168	171	
PTX3 (ng/mL)	15.4 ± 1.3	2.4 ± 0.25	**.002**
AFP (ng/mL)	1027.2 ± 32.7	3.8 ± 0.13	<**.0001**
CA199 (U/mL)	582 ± 24.7	12.1 ± 1.23	<**.0001**

Note: Statistical significance for continuous variables was assessed using an independent-sample *t*-test, while a chi-square test was applied to categorical variables. Results with *P* < .05 were deemed statistically significant and highlighted in bold text.

### 3.2. The expression of PTX3 in liver cancer tumor tissues and adjacent normal tissues

**Figure 1.**
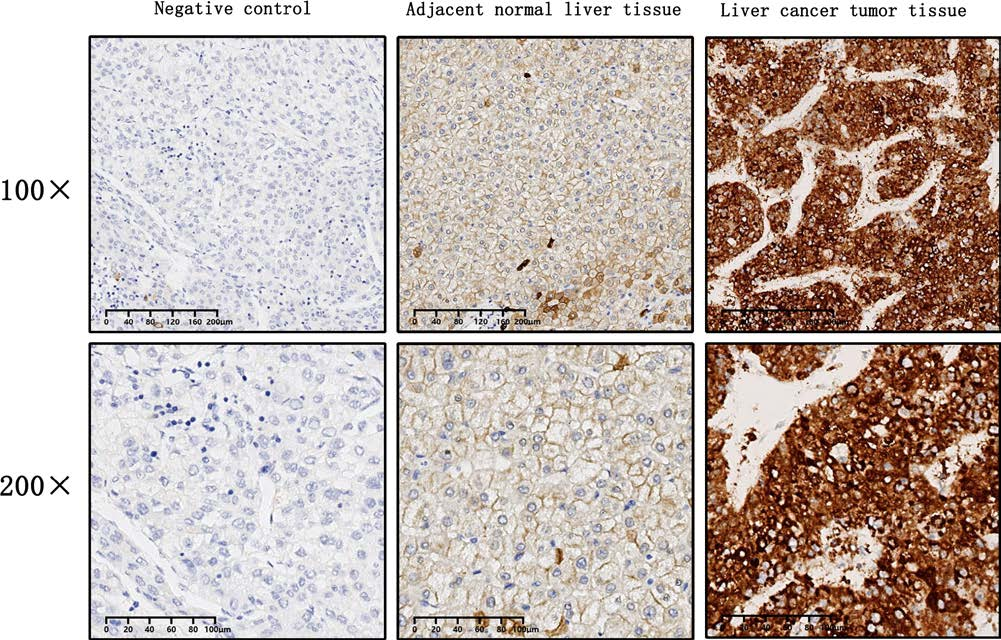
The expression of PTX3 in liver cancer tumor tissues and adjacent normal tissues.

**Figure 2.**
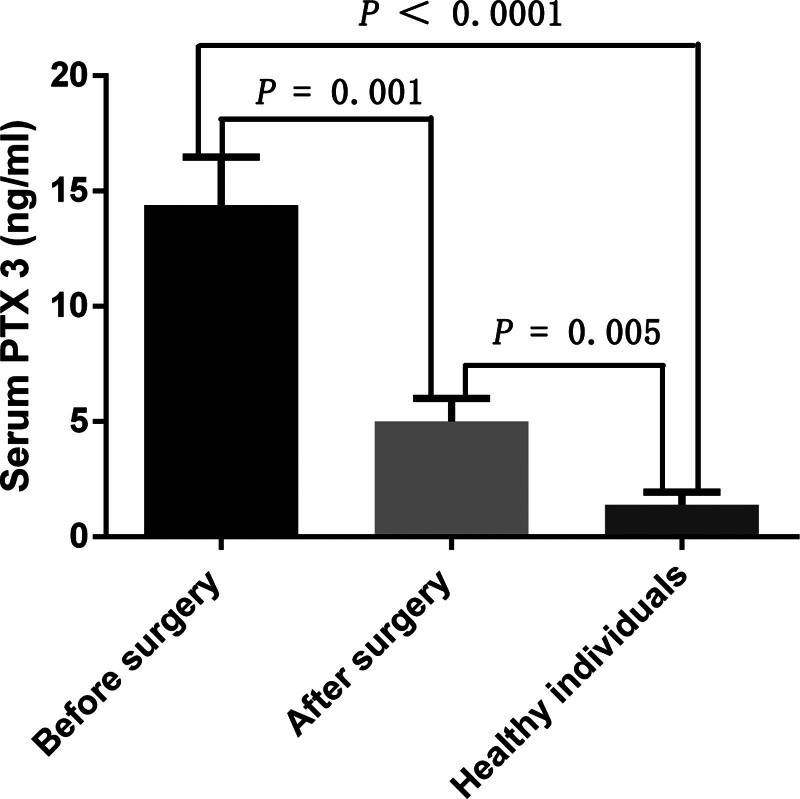
Serum PTX3 levels before and after surgery.

PTX3 expression was significantly higher in liver cancer tumor tissues compared to adjacent normal tissues (Fig. 1). To determine whether serum PTX3 originated from liver cancer tumor tissues, we measured serum PTX3 concentrations before and after the surgical removal of liver cancer tumors. The results demonstrated a significant decrease in PTX3 levels 1-week postsurgery (Fig. 2). Nevertheless, PTX3 levels remained significantly higher than those in healthy control subjects (Fig. 2).

### 3.3. Correlations of serum PTX3 levels with clinicopathological characteristics of liver cancer patients

Patients with liver cancer were divided into 2 groups based on the median serum PTX3 levels. The analysis revealed that PTX3 levels were significantly correlated with drinking history, TNM stage, BCLC stage, tumor size, tumor number, and vascular invasion (Table 2). However, no significant correlations were found between PTX3 levels and other factors, such as age, gender, BMI, liver cirrhosis, histological grade, and histological type (Table 2).

**Table 2:** Correlations of serum PTX3 levels with clinicopathological characteristics of liver cancer patients.

Characteristics	Total (180)	PTX3 levels		*P*-value
		Low (82)	High (98)	
Age (yr)				
≤60	75	31	44	.336
>60	105	51	54	
Gender				
Male	127	58	69	.962
Female	53	24	29	
BMI (kg/m^2^)				
≤26	98	42	56	.427
>26	82	40	42	
Drinking history				
No	54	34	20	**.002**
Yes	126	48	78	
Liver cirrhosis				
No	58	24	34	.438
Yes	122	58	64	
TNM stage				
I to II	95	35	60	**.0131**
III to IV	85	47	38	
BCLC stage				
A to B	102	38	64	**.011**
C to D	78	44	34	
Tumor size (cm)				
<5	104	36	68	**.0006**
≥5	76	46	30	
Tumor number				
Solitary	107	62	45	**.0001**
Multiple	73	20	53	
Histological grade				
G1 to G2	97	44	53	.955
G3 to G4	83	38	45	
Histological type				
Hepatocellular carcinoma	144	68	76	.369
intrahepatic cholangiocarcinoma	36	14	22	
Vascular invasion				
No	103	55	48	**.015**
Yes	77	27	50	

Note: A chi-square test analyzed the statistical significance. A *P* < .05 was considered statistically significant and highlighted in bold text.

### 3.4. Diagnostic performance of PTX3 in identifying liver cancer

**Figure 3.**
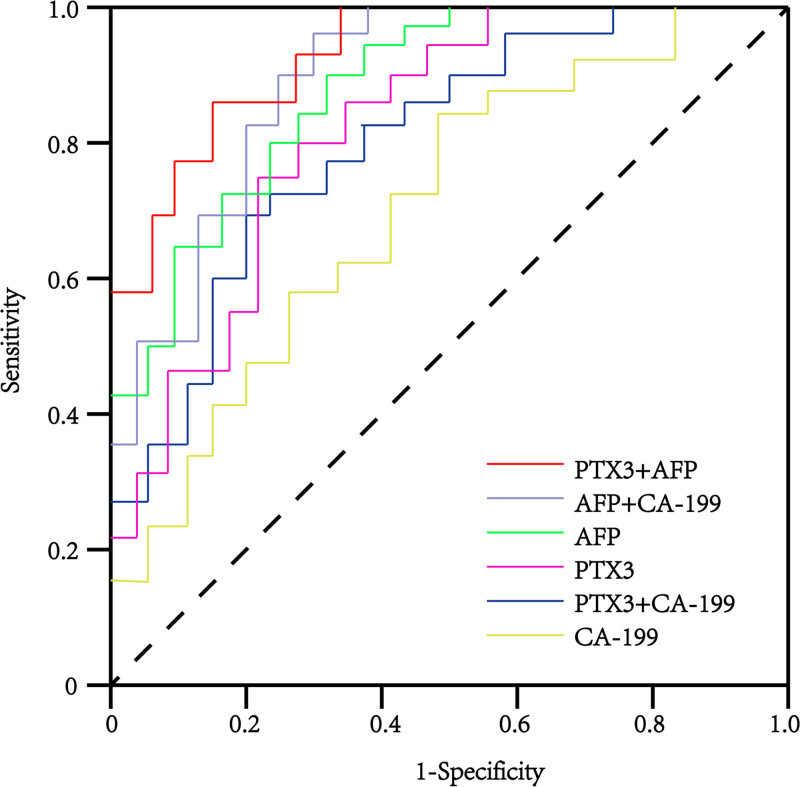
Diagnostic performance of PTX3 in identifying liver cancer.

The diagnostic performance of PTX3 was assessed using ROC curve analysis and compared with the liver cancer tumor markers AFP and CA199. PTX3 was able to differentiate liver cancer patients from healthy control subjects with an AUC of 0.734, a sensitivity of 73.24%, and a specificity of 84.78% (95% CI: 0.531–0.892) (Table 3 and Fig. 3). In comparison, AFP and CA199 levels showed AUCs of 0.813 (95% CI: 0.524–0.926) and 0.685 (95% CI: 0.516–0.827), respectively (Table 3 and Fig. 3). Notably, CA199 exhibited a specificity of <50%, underscoring its potential for high false-positive rates when used alone for liver cancer diagnosis. However, the combined use of AFP and PTX3 emerged as the most effective diagnostic strategy, achieving a sensitivity of 93.54%, specificity of 95.27%, and an AUC of 0.931 (95% CI: 0.727–0.983) (Table 3 and Fig. 3).

**Table 3:** Diagnostic performance of PTX3 in identifying liver cancer.

Biomarkers	AUC (95% CI)	Sensitivity (%)	Specificity (%)	Cutoff value	PPV (%)	NPV (%)	*P*-value
PTX3	0.734 (0.531–0.892)	73.24	84.78	5.1 ng/mL	84.3	87.2	**.002**
AFP	0.813 (0.524–0.926)	75.31	86.24	8.1 ng/mL	77.5	91.4	**.011**
CA199	0.685 (0.516–0.827)	69.27	38.87	37 U/mL	72.13	56.23	**.001**
PTX3 + AFP	0.931 (0.727–0.983)	93.54	95.27	–	95.78	94.12	**.014**
PTX3 + CA199	0.725 (0.431–0.824)	72.23	74.31	–	61.25	63.41	**.003**
AFP + CA199	0.842 (0.517–0.915)	78.29	88.34	–	83.25	85.13	**.015**

Notes: The receiver operating characteristic curve (ROC) and area under the curve (AUC) were used to assess the sensitivity, specificity, positive predictive value (PPV), negative predictive value (NPV), and cut-off value. A *P* < .05 was considered statistically significant and highlighted in bold text.

### 3.5. PTX3 as a predictor of poor prognosis in liver cancer patients

**Figure 4.**
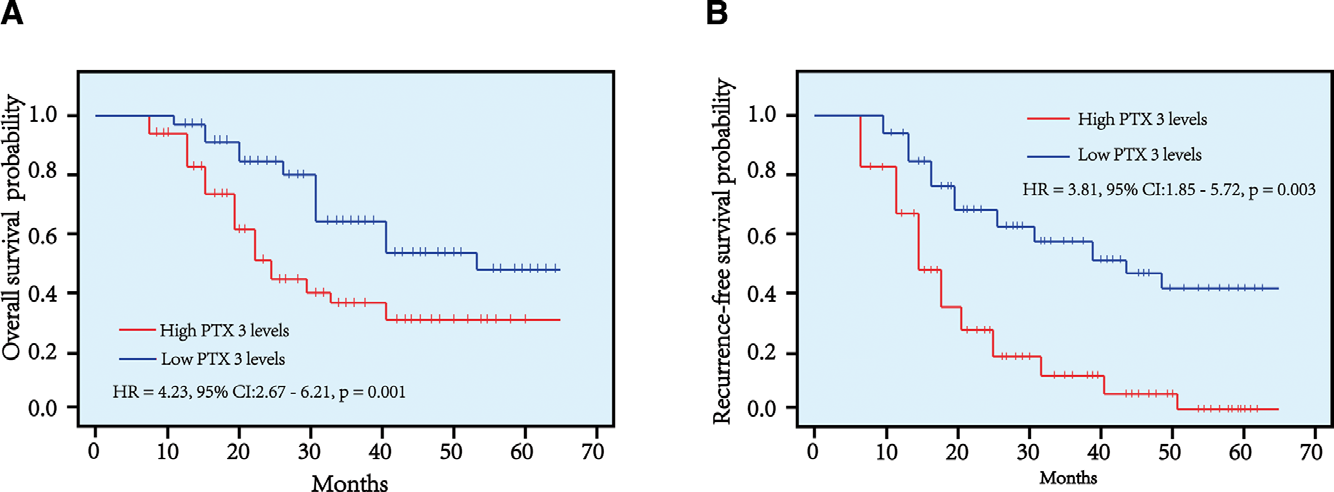
The overall survival and recurrence-free survival rate between patients with lower PTX3 levels and higher PTX3 levels.

Kaplan–Meier survival curves were generated to evaluate the prognostic significance of PTX3 for overall survival. The analysis indicated that patients with higher PTX3 levels had significantly poorer overall survival compared to those with lower PTX3 levels (HR: 4.23, 95% CI: 2.67–6.21, *P* = .001; Fig. 4A). Furthermore, recurrence-free survival was also assessed, and the results revealed that patients with elevated PTX3 levels experienced significantly worse RFS than those with lower PTX3 levels (HR: 3.81, 95% CI: 1.85–5.72, *P* = .003; Fig. 4B).

## 4. Discussion

According to global cancer statistics released by the World Health Organization (WHO) in 2022, liver cancer was the third leading cause of cancer-related death, responsible for 750,000 deaths, and the sixth most frequently diagnosed cancer, with over 860,000 new cases.^[1]^ Early detection of liver cancer is challenging due to the lack of clear symptoms in its early stages, often resulting in delayed diagnosis and treatment.^[4,19]^ By the time of diagnosis, many patients with liver cancer have already progressed to advanced stages, with some patients experiencing metastasis, which significantly impacts their prognosis.^[20,21]^ Early diagnosis allows for complete tumor resection, whereas patients diagnosed at late stages require comprehensive treatment strategies, including surgery, radiotherapy, and chemotherapy. However, their prognosis remains generally poor.^[22–24]^ Therefore, identifying biomarkers for early diagnosis is crucial for improving the survival outcomes of liver cancer patients.

PTX3, a member of the long pentraxin superfamily, is produced by immune cells and plays an important role in the response to pro-inflammatory cytokines, as well as in tissue repair and remodeling.^[25,26]^ PTX3 acts as a complement activator and aids in the recognition of phagocytic pathogens.^[27]^ However, recent research has emphasized its significant impact on tumor metastasis and the poor prognosis of cancers such as breast, lung, cervical, and ovarian cancers.^[11,13,14]^ PTX3 knockdown has been shown to reduce tumorigenicity and metastasis in human cervical cancer cells.^[28]^ Studies have also indicated that serum PTX3 levels are significantly higher in patients with lung cancer than in healthy individuals. Moreover, PTX3 is overexpressed in ovarian cancer, where high PTX3 expression correlates with poor prognosis in ovarian cancer patients.^[13]^ Nevertheless, the specific roles and clinical significance of PTX3 in liver cancer remain largely unknown.

This study revealed that serum PTX3 levels were elevated in patients with liver cancer compared to the healthy controls (Table 1). However, the specific mechanism underlying the increased serum PTX3 levels in these patients remains unclear. Notably, serum PTX3 levels significantly decreased following surgical removal of the liver tumor, suggesting that the elevated PTX3 levels may originate from liver tumor tissues (Fig. 2). Additionally, serum PTX3 levels remained markedly higher than those in healthy control subjects, even after tumor removal (Fig. 2). This persistent elevation could be attributed to the short 1-week interval between tumor resection and the measurement of serum PTX3 in postoperative patients, which may not have allowed sufficient time for PTX3 to degrade completely in the bloodstream. Alternatively, the sustained elevation of serum PTX3 could be due to circulating tumor cells that have not been entirely eliminated by either chemotherapy or the immune system. Future studies should focus on determining the timeframe needed for postoperative serum PTX3 levels to return to normal, as this could provide important information for monitoring liver cancer recurrence.

Although AFP and CA199 are commonly used biomarkers for diagnosing liver cancer, their sensitivity and specificity are not optimal.^[29]^ AFP lacks sufficient sensitivity for intrahepatic cholangiocarcinoma, while CA199 is not specific to liver cancer, leading to a high risk of false positives.^[30,31]^ Our study demonstrated that PTX3 is a reliable and promising biomarker for diagnosing liver cancer, with an AUC of 0.734, a sensitivity of 73.24%, and a specificity of 84.78% (Table 3 and Fig. 3). Moreover, the combination of PTX3 with AFP provided the most effective diagnostic approach, achieving an AUC of 0.931, a sensitivity of 93.54%, and a specificity of 95.27% (Table 3 and Fig. 3). These findings suggest that PTX3 can significantly enhance the early diagnosis of liver cancer, which is essential for providing timely treatment and improving patient outcomes. However, further research is needed to confirm these findings in a larger and more diverse patient population.

The recurrence rate of liver cancer is particularly high, especially in advanced cases.^[32,33]^ It is widely acknowledged that factors such as tumor differentiation, tumor size, lymph node metastasis, and pathological stage are associated with the recurrence of liver cancer.^[34,35]^ Our study showed that serum PTX3 levels were significantly correlated with TNM stage, tumor size, and lymph node metastasis in liver cancer (Table 2). This suggests that serum PTX3 has the potential to be a valuable biomarker for predicting liver cancer recurrence. Additionally, liver cancer patients with higher serum PTX3 levels exhibited lower overall survival and RFS rates compared to those with lower levels (Fig. 4A and B). These results imply that serum PTX3 could serve as a prognostic biomarker for liver cancer patients. Therefore, patients with elevated serum PTX3 levels should undergo vigilant postsurgical monitoring to detect any signs of recurrence. Neglecting these indicators could result in delayed treatment and a poor prognosis.

Our study has several strengths. First, this is the first study to evaluate the potential of serum PTX3 as a biomarker for both the diagnosis and prognosis of primary liver cancer, potentially advancing diagnostic and treatment strategies. Second, all laboratory and clinical assessments were conducted using standardized and highly reliable procedures. Third, all participants were newly diagnosed liver cancer patients who had not received any prior medical treatment, thus minimizing the impact of confounding factors. However, there are some limitations to this study. First, we did not explore the specific mechanisms responsible for elevated serum PTX3 levels in liver cancer patients. Second, the sample size was relatively small and limited to the Chinese population, which may affect the generalizability of the results to broader populations. Third, despite employing methods such as stratification, matching, and well-defined defined inclusion and exclusion criteria to minimize selection bias, we could not entirely eliminate it.

## 5. Conclusions

Serum PTX3 levels are elevated in liver cancer patients and high serum PTX3 levels are associated with poor prognosis. This suggests that serum PTX3 has potential as a novel biomarker for both the diagnosis and prognosis of liver cancer. These findings may improve patient outcomes by enabling early detection and continuous monitoring. However, further multi-center clinical studies are needed to validate the use of serum PTX3 as a reliable biomarker for the diagnosis and prognosis of primary liver cancer and to explore the specific mechanisms responsible for the increased serum PTX3 levels in liver cancer patients.

## Acknowledgments

We extend our thanks to all patients who participated in this study.

## Author contributions

**Conceptualization:** Hongchi Zhou.

**Funding acquisition:** Mao Chen.

**Investigation:** Li Chen, Hongchi Zhou.

**Methodology:** Li Chen, Mao Chen, Qin Liu.

**Project administration:** Mao Chen.

**Resources:** Shifu Song.

**Software:** Shifu Song.

**Validation:** Qin Liu.

**Writing – original draft:** Li Chen, Qin Liu.

**Writing – review & editing:** Li Chen, Qin Liu.
